# Curcumin Reduces Pathological Endoplasmic Reticulum Stress through Increasing Proteolysis of Mutant Matrilin-3

**DOI:** 10.3390/ijms24021496

**Published:** 2023-01-12

**Authors:** Ella P. Dennis, Robyn N. Watson, Florence McPate, Michael D. Briggs

**Affiliations:** International Centre for Life, Biosciences Institute, Newcastle University, Newcastle upon Tyne NE1 3BZ, UK

**Keywords:** genetic skeletal disease, chondrodysplasia, matrilin-3, endoplasmic reticulum (ER) stress, curcumin, proteasomal degradation

## Abstract

The intracellular retention of mutant cartilage matrix proteins and pathological endoplasmic reticulum (ER) stress disrupts ossification and has been identified as a shared disease mechanism in a range of skeletal dysplasias including short limbed-dwarfism, multiple epiphyseal dysplasia type 5 (EDM5). Although targeting ER stress is an attractive avenue for treatment and has proven successful in the treatment of a related skeletal dysplasia, to date no drugs have proven successful in reducing ER stress in EDM5 caused by the retention of mutant matrilin-3. Our exciting findings show that by using our established luciferase ER stress screening assay, we can identify a “natural” chemical, curcumin, which is able to reduce pathological ER stress in a cell model of EDM5 by promoting the proteasomal degradation mutant matrilin-3. Therefore, this is an important in vitro study in which we describe, for the first time, the success of a naturally occurring chemical as a potential treatment for this currently incurable rare skeletal disease. As studies show that curcumin can be used as a potential treatment for range of diseases in vitro, current research is focused on developing novel delivery strategies to enhance its bioavailability. This is an important and exciting area of research that will have significant clinical impact on a range of human diseases including the rare skeletal disease, EDM5.

## 1. Introduction

Autosomal dominant mutations in the *MATN3* gene, encoding the cartilage extracellular matrix protein matrilin-3, have been shown to cause the short-limbed dwarfism multiple epiphyseal dysplasia (MED) type 5 (EDM5; OMIM #607078) [1,2,3]. Such pathological mutations occur within the highly conserved von Willebrand factor A (vWFA) domain of matrilin-3 and disrupt protein folding. Mutant matrilin-3 is then retained within the endoplasmic reticulum (ER) where it forms supramolecular aggregates due to the exposure of unpaired cysteine residues [4,5,6,7]. This accumulation of mutant matrilin-3 induces pathological ER stress and the unfolded protein response (UPR) has detrimental consequences on chondrocyte phenotype (proliferation and apoptosis), ultimately impairing endochondral ossification and long bone formation [4,5,6,7].

Misfolded mutant protein retention and pathological ER stress causing the UPR has been identified as a common disease mechanism in several forms of skeletal dysplasia including MED and metaphyseal chondrodysplasia type Schmid caused by mutations in the gene encoding type X collagen (*COL10A1*) (MCDS, OMIM # 156500) [3,8]. Therefore, since pathological ER stress is a unifying mechanism for these rare skeletal diseases, it has become an attractive therapeutic avenue. Indeed, recent studies show that the administration of the anti-epileptic drug carbamazepine (CBZ) promotes the degradation of mutant collagen X, reduces ER stress, ameliorates disease severity, and restores long bone growth [9,10]. Despite the success of CBZ in the treatment of MCDS, CBZ had no effect on ER stress caused by mutations in matrilin-3 and this has potentially been attributed to [3]. Since no drugs have proven successful in reducing ER stress caused by mutant matrilin-3 retention, we, therefore, sought to identify alternative compounds, with the ability of promoting mutant matrilin-3 degradation and reducing pathological ER stress, which could offer therapeutic potential for patients with EDM5.

## 2. Results

### 2.1. The Naturally Occurring Chemicals, Vitexin and Curcumin, Significantly Reduce Pathological ER Stress Caused by the Retention of Mutant Matrilin-3 without Affecting Wild-Type Cells

Based on the published literature, we identified three naturally occurring chemicals ((1) Gingko extract—prepared from the leaves of the Gingko biloba tree, (2) vitexin—a glycosylated flavonoid found in plant leaves, (3) curcumin—the principle curcuminoid of turmeric) that were able to reduce ER stress in in vitro and in vivo studies [11,12,13,14,15,16,17,18,19,20,21]. We have previously shown that pathological ER stress caused by the retention of mutant matrilin-3 can be successfully monitored in vitro using a luciferase ER stress reporter plasmid (ERSE1) [3]. As ER stress is an attractive target to treat skeletal dysplasias including EDM5, we, therefore, used our established luciferase screening assay to determine if these “natural” chemicals were able to reduce pathological ER stress in cells expressing the archetypal V194D matrilin-3 vWFA mutation.

**Figure 1:**
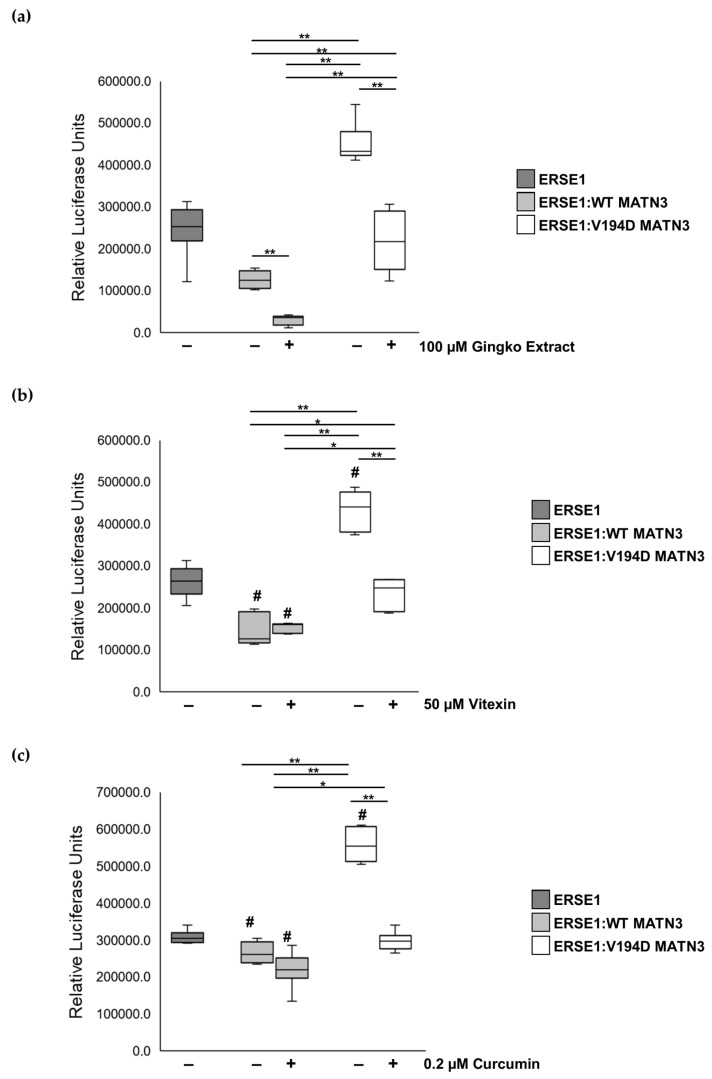
**Administration of curcumin and vitexin ameliorates ER stress caused by the retention of mutant matrilin-3.** HELA cells were co-transfected with the ER stress luciferase reporter plasmid (ERSE1) and either wild-type (ERSE1:WT MATN3) or V194D mutant matrilin-3 (ERSE1:V194D MATN3) and cells were cultured in the presence/absence of 100 µM Gingko extract, 50 µM vitexin, or 0.2 µM curcumin. ER stress was monitored using a luciferase assay. Mutant matrilin-3-induced ER stress in HELA cells was identified by increased luciferase activity. (**a**) Luciferase activity is significantly reduced in both ERSE1:WT MATN3 and ERSE1:V194D MATN3 cells following treatment with Gingko extract, indicating that Gingko extract not only significantly reduces ER stress in V194D mutant matrilin-3-expressing cells but it also disrupts ‘normal’ ER homeostasis in wild-type matrilin-3-expressing cells. (**b**) Vitexin and (**c**) curcumin do not affect ER stress/ER homeostasis in wild-type matrilin-3-expressing cells; however, both significantly reduce pathological ER stress in V194D mutant matrilin-3-expressing cells since luciferase activity returns to WT levels 48 hours after treatment. (ERSE1: ER stress reporter plasmid, MATN3: matrilin-3, WT: wild-type. *n* = 3. # = significant to ERSE1, * *p* < 0.05, ** *p* < 0.005).

Although luciferase activity is significantly reduced in cells expressing V194D mutant matrilin-3 (ERSE1:V194D MATN3) following treatment with gingko extract, we find that the administration of gingko extract also significantly reduces luciferase activity in wild-type matrilin-3-expressing cells (ERSE1:WT MATN3) (Figure 1A). Since luciferase activity is a measure of ER stress, gingko extract is discounted as a potential treatment for EDM5 since it also affects physiological ER stress in wild-type cells. Interestingly, treatment with vitexin or curcumin significantly reduces luciferase activity by 41.6-fold/45.5-fold, respectively, in ERSE1:V194D MATN3 cells, returning luciferase activity to levels comparable with ERSE1:WT MATN3 (Figure 1B,C). However, unlike the gingko extract that reduces physiological ER stress levels in wild-type matrilin-3-expressing cells, neither vitexin nor curcumin has any effect on luciferase activity and, therefore, ER homeostasis in ERSE1:WT MATN3 cells. These results, therefore, reveal that both vitexin and curcumin reduce the pathological ER stress caused by the retention and aggregation of mutant matrilin-3, with no detectable effect on wild-type cells.

**Figure 2:**
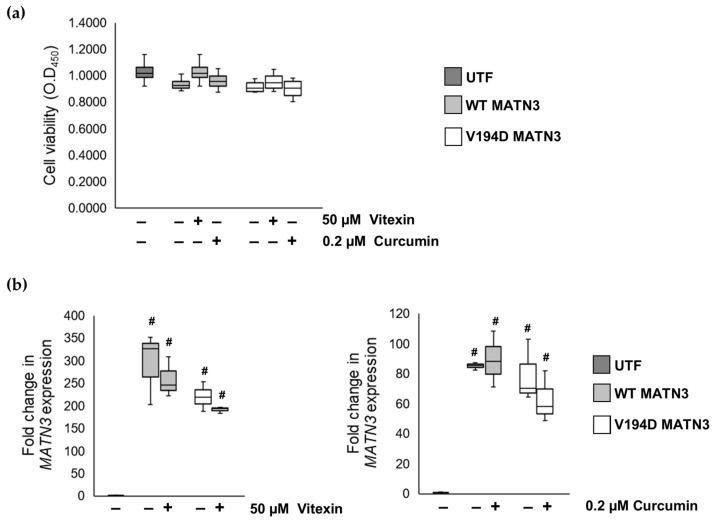
**The effects of curcumin and vitexin on reducing pathological ER stress in mutant matrilin-3-expressing cells were independent of reducing both cell viability and MATN3 expression.** WT and V194D mutant MATN3-expressing HELA cells were cultured in the presence/absence of either 50 µM vitexin or 0.2 µM curcumin for 48 h. (**a**) To assess if vitexin or curcumin affect cell viability, a WST1 assay is used, as WST1 absorbance is an indicator of cell viability. The administration of vitexin and curcumin to both WT and V194D mutant MATN3-expressing HELA cells does not significantly affect cell viability as absorbance is comparable between treated and untreated cells. (**b**) To determine the effects of treatment on gene expression, the expression level of the gene encoding matrilin-3, MANT3, was measured by qPCR. The administration of vitexin and curcumin to both WT and mutant V194D MATN3-expressing HELA cells does not significantly affect the expression of MATN3. (MATN3: matrilin-3, WT: wild-type, UTF: untransfected, # significant to UTF, *n* = 3).

To determine if the reduction in ER stress levels in ERSE1:V194D MATN3 cells cultured with either vitexin or curcumin was not due to increased cell death following treatment, cell viability was determined using a WST1 assay. Since neither vitexin nor curcumin treatment affects the viability of wild-type or V194D mutant matrilin-3-expressing cells (Figure 2), this indicates that the reduction in ER stress following treatment is not a result of these chemicals inducing cell death.

Although we determined that vitexin/curcumin treatment did not affect cell viability, we needed to ensure that the reduction in ER stress levels following treatment was not due to the drugs impairing *MATN3* expression. As HELA cells do not endogenously express the *MATN3* gene, *MATN3* expression identified by quantitative PCR (qPCR) was expressed exclusively from the exogenous transfected DNA template. Interestingly, qPCR reveals that neither vitexin nor curcumin treatment affects the expression of *MATN3,* as gene expression levels are comparable between treated and untreated cells (Figure 2). These results, therefore, show that the reduction in ER stress levels following vitexin/curcumin treatment is independent of impairing cell viability and gene expression, therefore, indicating that these natural chemicals could potentially have a direct effect on mutant protein retention.

### 2.2. Curcumin Treatment Reduces the Intracellular Accumulation of Mutant Matrilin-3 and Ameliorates Pathological ER Stress Signalling in a Cell Model of EDM5

**Figure 3:**
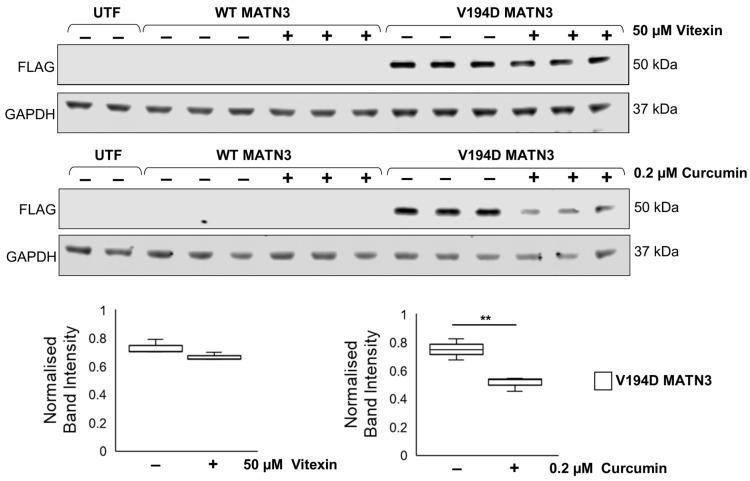
**Curcumin treatment reduces the intracellular accumulation of mutant matrilin-3.** WT and V194D mutant MATN3-expressing HELA cells were cultured in the presence/absence of either 50 µM vitexin or 0.2 µM curcumin for 48 h and the intracellular expression of FLAG-tagged matrilin-3 was analysed by Western blotting. Wild-type matrilin-3 was secreted from the cell and so was not detected in the cell lysate, however, V194D mutant matrilin-3 was retained intracellularly. Although vitexin has no effect on intracellular protein expression, curcumin treatment significantly reduces the accumulation of intracellular mutant matrilin-3. (Equal loading shown by GAPDH. FLAG: FLAG-tagged matrilin-3, GAPDH: glyceraldehyde-3-phosphate dehydrogenase, MATN3: matrilin-3, WT: wild-type, UTF: untransfected, ** *p* < 0.005, *n* = 3).

To determine if vitexin/curcumin treatment reduced pathological ER stress due to modulating the intracellular retention of V194D mutant matrilin-3, we assessed intracellular matrilin-3 protein levels in treated/untreated cells by Western blotting. Although gene expression analysis reveals that WT MATN3 cells successfully express wild-type *MATN3*, because matrilin-3 is a secreted cartilage extracellular matrix protein its expression is not detectable in the cell lysate unlike V194D mutant matrilin-3 that is retained and detected within the cell (Figure 3). Interestingly, treatment with 50 µM vitexin has no effect on the intracellular levels of V194D mutant matrilin-3; however, 0.2 µM curcumin treatment significantly reduces the intracellular expression of the mutant protein by 31.65% (Figure 3). Based on published reports linking curcumin to protein degradation [22,23], these data suggest that curcumin treatment could potentially promote the degradation of mutant matrilin-3.

**Figure 4:**
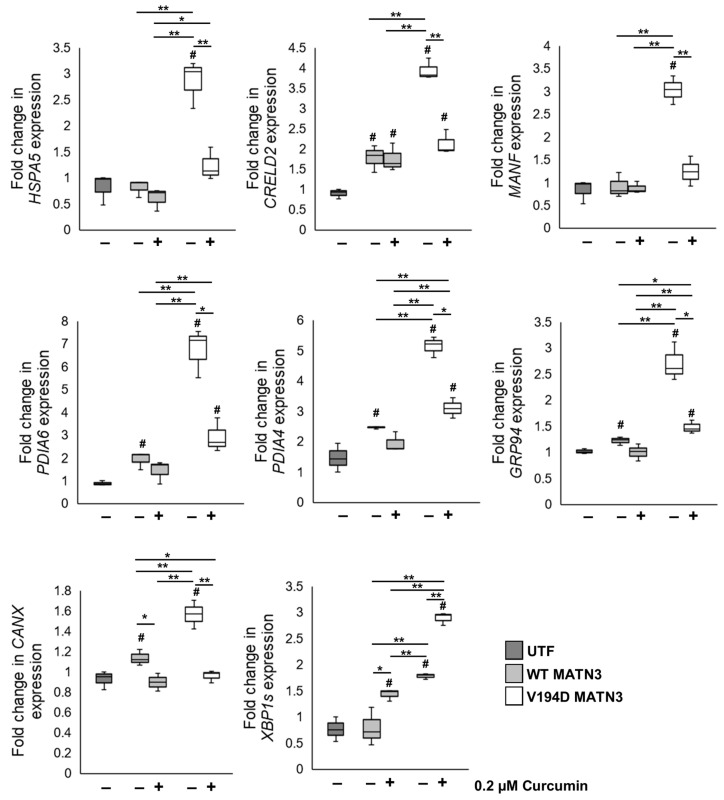
**Although curcumin reduces the expression of UPR marker genes, it promotes XBP1 splicing.** WT and V194D mutant MATN3-expressing HELA cells were cultured in the presence/absence of 0.2 µM curcumin for 48 h and the expression of UPR marker genes were analysed by qPCR. HSPA5, encoding the master regulator of the UPR BiP, is significantly downregulated in V194D MATN3 cells treated with curcumin. In addition, genes downstream of ATF6/XBP1, including CRELD2, MANF, PDIA6, PDIA4, GRP94, and CANX, are also significantly downregulated in mutant matrilin-3-expressing cells. Interestingly, XBP1 splicing is upregulated following curcumin treatment. (Expression normalised to 18S levels. MATN3: matrilin-3, WT: wild-type, UTF: untransfected, # significant to UTF, * *p* < 0.05, ** *p* < 0.005, *n* = 3).

We have previously shown that the retention and accumulation of V194D mutant matrilin-3 results in the pathological activation of the ATF6 and IRE1/XBP1 branch of the UPR [6,7,24]. Here, we confirm that the intracellular retention of V194D mutant matrilin-3 promotes the splicing of XBP1 (XBP1s), the downstream effector of active IRE1 signalling, and results in the upregulation key URP genes including HSPA5, encoding binding immunoglobulin protein (BiP), the master regulator of the UPR, as well as genes encoding several ATF6/XBP1s regulated chaperones such as MANF, PDIA4, CANX, PDIA6, GRP94, and CRELD2 (Figure 4).

As several published studies show that curcumin treatment reduces ER stress in a range of conditions [13,16,17,19,21], using qPCR we study the expression of UPR genes upregulated in our mutant matrilin-3 cell model of EDM5 following treatment with curcumin. Our results show that, with the exception of XBP1s, all UPR marker genes are significantly downregulated in V194D mutant matrilin-3-expressing cells treated with curcumin. For example, HSPA5, CRELD2, MANF, and PDIA6 levels are significantly reduced in V194D MATN3, to levels comparable with those seen in WT MATN3 cells. In addition, although the expression levels of PDIA4, GRP94, and CANX are significantly upregulated in curcumin-treated V194D MATN3 cells relative to WT MATN3 cells, these genes are still significantly downregulated in treated V194D MATN3 cells compared to untreated V194D mutant matrilin-3-expressing cells by 39.66%, 45.43%, and 38.57%, respectively. Interestingly, the only gene that is not downregulated following curcumin treatment was XBP1s, which is, in fact, upregulated 5.71-fold in curcumin-treated V194D MATN3 cells.

### 2.3. Curcumin Promotes Proteolysis of Mutant Matrilin-3

It is well-documented that XBP1s functions to maintain ER homeostasis by upregulating molecular chaperones, folding enzymes and components of the ER-associated degradation (ERAD) pathway that function to eliminate misfolded proteins from the ER by targeting them for degradation by the proteasome [25]. A recent study highlights that modulation of this important ERAD pathway is proven to be successful in the treatment of a related chondrodysplasia, metaphyseal chondrodysplasia type Schmid type (MCDS) [9]. Here, the authors demonstrate that CBZ treatment ameliorates pathological ER stress by promoting proteasomal and/or lysosomal degradation of mutant type X collagen.

**Figure 5:**
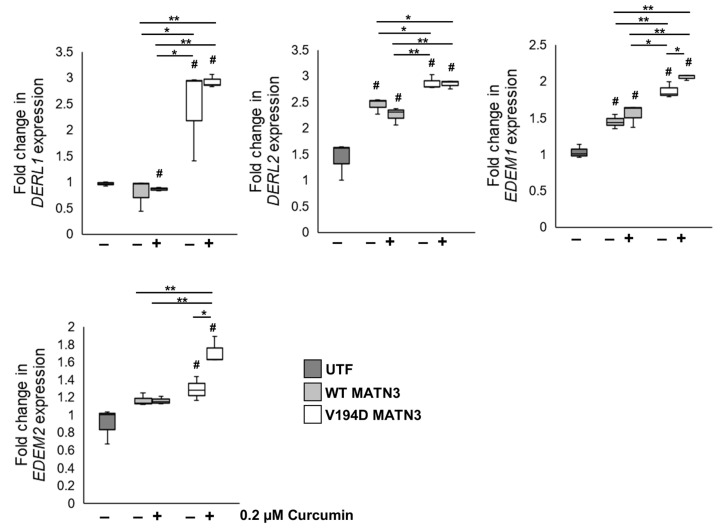
**Curcumin promotes the expression of ERAD genes.** WT and V194D mutant MATN3-expressing HELA cells were cultured in the presence/absence of 0.2 µM curcumin for 48 h and the expression of ERAD marker genes were analysed by qPCR. Although curcumin treatment of V194D cells significantly upregulates genes encoding EDEM proteins (EDEM1 and EDEM2) that function to recognise and target misfolded proteins for proteasomal degradation, it has no effect on the expression of genes encoding Derlin membrane proteins (DERL1 and DERL2) that function as part of the ER retro-translocon machinery. (Expression normalised to 18S levels. MATN3: matrilin-3, WT: wild-type, UTF: untransfected, # significant to UTF, * *p* < 0.05, ** *p* < 0.005, *n* = 3).

As curcumin treatment upregulates XBP1 splicing and reduces the intracellular expression of mutant matrlin-3 in vitro, we studied the effects of curcumin treatment on the expression of ERAD genes in our cell model of EDM5. Specifically, we analyse the expression of members of genes encoding the ER degradation-enhancing alpha-mannosidase-like protein (EDEM) family (EDEM1 and EDEM2), which function to recognise and target misfolded glycoproteins for retro-translocation from the ER lumen [26,27,28,29]. In addition, we studied the expression of a second family of genes (DERL1 and DERL2) encoding Derlin membrane proteins that function as part of the retro-translocation machinery [30,31,32]. Although there is no significant difference in the expression of Derlin genes between untreated and curcumin-treated V194D MATN3 cells (Figure 5), EDEM1 and EDEM2 are significantly upregulated in V194D mutant matrilin-3-expressing cells following curcumin treatment. Based on these findings, we, therefore, hypothesised that following curcumin treatment, mutant matrilin-3 is targeted for proteasomal degradation by the EDEM1/2-mediated ERAD pathway.

**Figure 6:**
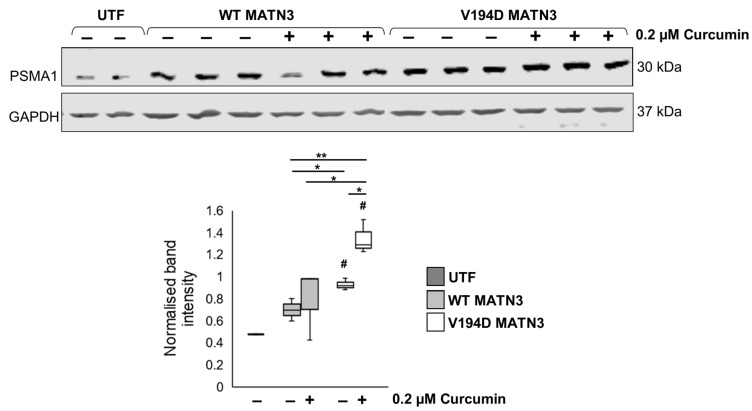
**Curcumin treatment upregulates proteasome expression in mutant matrilin-3-expressing cells.** WT and V194D mutant MATN3-expressing HELA cells were cultured in the presence/absence of 0.2 µM curcumin for 48 h and the intracellular expression of the proteasomal subunit PSMA1 was analysed by Western blotting. PSMA1 levels are unaffected by curcumin treatment in WT MATN3 cells; however, administration of curcumin promotes the upregulation of PSMA1 in V194D MATN3 cells. (Equal loading shown by GAPDH. PSMA1: proteasomal subunit alpha-type 1, GAPDH: glyceraldehyde-3-phosphate dehydrogenase, MATN3: matrilin-3, WT: wild-type, UTF: untransfected, # significant to UTF, * *p* < 0.05, ** *p* < 0.005, *n* = 3).

Since curcumin has been shown to result in the upregulation of proteasomal genes [33] and our data suggest that mutant matrilin-3 is potentially targeted for degradation by the proteasome following curcumin treatment, we analyse the expression of an essential subunit of the 20S core of the proteasome complex, proteasome subunit alpha-type 1 (PSMA1), by Western blotting. PSMA1 is upregulated 1.33-fold in V194D mutant matrilin-3-expressing cells relative to cells expressing wild-type matrilin-3. Although curcumin does not have any effect on the expression of the PSMA1 proteasome subunit in wild-type cells, treatment with curcumin results in a significant upregulation of PSMA1 in V194D MATN3 cells (Figure 6). More specifically, PSMA1 is upregulated 1.93-fold and 1.45-fold in curcumin-treated V194D cells relative to WT MATN3 cells and untreated V194D MATN3 cells, respectively. Taken together, these findings strengthen our hypothesis that curcumin treatment promotes proteasomal degradation of mutant matrilin-3.

**Figure 7:**
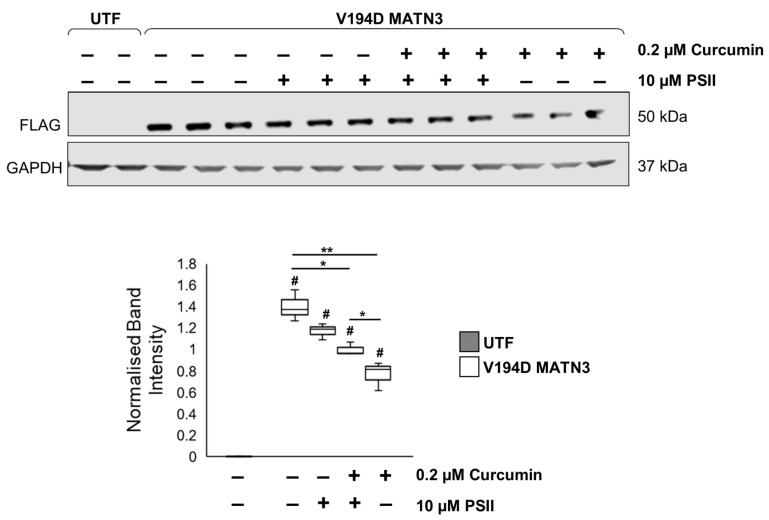
**Curcumin promotes proteasomal degradation of V194D mutant matrilin-3 in a cell model of EDM5.** V194D mutant MATN3-expressing HELA cells were cultured in the presence/absence of 0.2 µM curcumin for 48 h. To study if curcumin promotes the proteasomal degradation of mutant matrilin-3, the proteasomal inhibitor PSII is added to block the actions of the proteasome. After 48 h, the intracellular expression of FLAG-tagged matrilin-3 was analysed by Western blotting. Curcumin treatment significantly reduces the accumulation of intracellular mutant matrilin-3. PSII administration blocks the effects of curcumin and results in an upregulation in the intracellular expression of mutant matrilin-3 to levels comparable to untreated cells, confirming that curcumin promotes the proteolysis of mutant matrilin-3 by the proteasome. (Equal loading shown by GAPDH. FLAG: FLAG-tagged matrilin-3, GAPDH: glyceraldehyde-3-phosphate dehydrogenase, MATN3: matrilin-3, PSII: proteasome inhibitor II, WT: wild-type, UTF: untransfected, # significant to UTF, * *p* < 0.05, ** *p* < 0.005, *n* = 3).

To confirm if mutant matrilin-3 is degraded by the proteasome following the administration of curcumin, a proteasome inhibitor, PSII, is added to block the effects of curcumin. As observed in Figure 3, curcumin treatment significantly reduces the intracellular expression of mutant matrilin-3 (Figure 7). Interestingly, this reduction in mutant matrilin-3 expression following treatment with curcumin is prevented upon PSII administration. Indeed, mutant matrilin-3 is upregulated 1.31-fold in V194D MATN3 cells treated with both curcumin and PSII relative to cells treated with curcumin alone. In fact, the levels of intracellular mutant matrilin-3 expression are comparable between untreated V194D cells and V194D cells treated with both curcumin and PSII. These results, therefore, show, for the first time, that curcumin promotes the proteasomal degradation of V194D mutant matrilin-3, subsequently ameliorating pathological ER stress, and highlights the potential for this naturally occurring chemical as a treatment for EDM5.

## 3. Discussion and Conclusions

Pathological ER stress due to the intracellular retention of a mutant protein is a common mechanism for a range of diseases that affect long bone growth and development [9,34]. As such, the administration of chemical chaperones has proven successful at restoring ER homeostasis in the treatment of rare skeletal conditions [9,34]. Despite the success of these chemicals in ameliorating disease-causing ER stress, no ER-stress-modifying chemicals have proven successful in the treatment of EDM5 [3,6]. Here, we describe, for the first time, the success of a naturally occurring chemical as a potential treatment for this currently incurable rare skeletal disease.

Natural chemicals have been central to the treatment of many pathological conditions, as many approved drugs are obtained from plants or designed as synthetic derivatives of natural products. For example, the nonsteroidal anti-inflammatory drug aspirin, and the widely used chemotherapy drug pacitaxel, are derived from willow tree bark and the bark of the pacific yew tree, respectively [35]. In this study, we identified three naturally occurring ER-stress-modifying chemicals (Gingko extract, vitexin, and curcumin) and tested their potential at reducing pathological ER stress in our cell model of EDM5 [11,12,13,14,15,16,17,18,19,20,21].

In agreement with previous studies, we find that Gingko extract is able to reduce pathological ER stress levels in our V194D mutant matrilin-3 cell model of EDM5 [11,12,15]. Despite this promising finding, Gingko extract also reduces ER stress levels in wild type cells and, therefore, affects physiological ER homeostasis. As such, Gingko extract is discounted as a potential treatment for EDM5. On the other hand, our findings reveal that both vitexin and curcumin are also able to reduce pathological ER stress in V194D-expressing cells without affecting wild-type cell homeostasis.

Since the intracellular retention and accumulation of mutant matrilin-3 results in the co-retention of other cartilage extracellular matrix proteins, it is important that any potential treatment for EDM5 not only reduces pathological ER stress, but also promotes the degradation of mutant matrilin-3 [36]. Indeed, CBZ proved successful in the treatment of a related skeletal dysplasia, as it promoted the proteolysis of retained mutant collagen X [9]. Although vitexin reduces pathological ER stress signalling in V194D MATN3 cells, it has no effect on the retention of mutant matrilin-3 and is, therefore, also discounted as a potential therapeutic agent for the treatment of EDM5. We attribute this finding to the inhibitory effect of vitexin on polyubiquitin synthesis, a key step required for targeting proteins for proteasomal degradation [37]. Despite the disappointing outcomes of gingko extract/vitexin treatment, our exciting results reveal that curcumin administration successfully reduces pathological ER stress by promoting proteasomal degradation of V194D mutant matrilin-3.

Our recently published paper highlights the importance of spliced XBP1 signalling in EDM5 because knocking out cartilage XBP1 expression in our mouse model of matrilin-3 MED exacerbates the disease phenotype [24]. In line with a previous study, here we show that curcumin treatment promotes protective XBP1 splicing in V194D mutant matrilin-3-expressing cells, which results in a significant upregulation of XBP1s target genes [21]. These upregulated XBP1s target genes include EDEM1 and EDEM2, which encode ERAD molecular chaperones important for extracting misfolded glycoproteins from the calnexin cycle and targeting them for proteasomal degradation [38,39]. As reported previously, the results presented here show that curcumin treatment not only upregulates these EDEM genes, but it also results in a reduction in the expression of the lectin chaperone CANX, which functions to ensure the proper folding of glycoproteins such as matrilin-3 [40,41]. It is important to note that although several published studies show that curcumin inhibits proteasome activity, this effect appears to be dose-dependent and low doses of curcumin are shown to actually activate proteasome activity [22,23,42,43,44,45]. In accordance with several studies, our results reveal that curcumin treatment upregulates the proteasome, as indicated by an upregulation in a key subunit of the proteasome core, and by using a proteasome inhibitor we confirmed that mutant matrilin-3 is degraded by the proteasome following curcumin treatment [33]. This study, therefore, suggests that in our cell model of EDM5, curcumin promotes XBP1s-mediated ERAD, allowing misfolded mutant matrilin-3 to escape the calnexin cycle and be targeted for degradation by the proteasome, which, in turn, reduces pathological ER stress.

To date, several studies have highlighted curcumin as a potential effective treatment for a range of chronic diseases [46]. Despite these encouraging findings, curcumin has not been approved as a therapeutic drug. Although it has a good safety record in vitro, this has not been supported by clinical data. In addition, a major issue with curcumin as a therapeutic agent is its extremely low bioavailability, which has been attributed to its chemical instability, poor absorption, and rapid metabolism and elimination [47]. In fact, studies have shown that circulating curcumin levels in the blood were extremely low and even undetectable in some cases. Therefore, although our exciting findings highlight curcumin as a potential treatment for EDM5 in vitro, this would not be easily translatable to in vivo. For example, the dose of curcumin must be tightly controlled to have the desired effect on activating the proteasome while not compromising cell viability and activity [48]. Not only does the low bioavailability of curcumin pose a problem when treating EDM5, but since cartilage is an avascular, a lymphatic, and anerual tissue, it is extremely difficult to target drugs to cartilage growth plate forming chondrocytes.

To summarise, our results propose that curcumin may provide a novel therapeutic avenue for the treatment of EDM5, as it promotes the proteolysis of retained mutant matrilin-3, thereby reducing pathological ER stress. In order for curcumin to become an approved therapeutic agent, current research is focusing on novel delivery strategies and enhancing its bioavailability. Due to the exciting therapeutic potential of this natural chemical, this is an extremely important goal for research that will impact the treatment of a wide range of human conditions, including the rare skeletal disease EDM5.

## 4. Materials and Methods

### 4.1. Cell Culture

HeLa cells were previously transfected with an ER stress luciferase reporter plasmid that was kindly gifted from Seiichi Oyadomari (pGL4-ERSE1-luc2P-Hygro, Addgene plasmid #101789). ERSE1 cells were cultured in complete DMEM supplemented with 10% FBS, 5% non-essential amino-acids, 1 U/mL penicillin, 1 μg/mL streptomycin, and 150 μg/mL hygromycin B (all from Thermo Fisher, Waltham, MA, USA). Lipofectamine 2000 (Thermo Fisher, Waltham, MA, USA) was used to transiently transfect either untransfected (UTF) HELA cells or ERSE1 cells with previously generated plasmids expressing either human wild-type (WT MATN3) or V194D mutant matrilin-3 (V194D MATN3) [7]. Transfected cells were then treated with either 100 µM gingko extract, 50 µM vitexin, or 0.2 µM curcumin (all from Sigma Aldrich, Burlington, MA, USA) 30 min after transfection, and cells were cultured for a period of 48 h. To assess proteasomal degradation, transfected cells were cultured in the presence/absence of curcumin and 10 µM proteasome inhibitor PSII (Sigma Aldrich, Burlington, MA, USA) was added for the final 8 h of culture.

### 4.2. Luciferase Assay

ERSE1 cells expressing either wild-type or V194D mutant matrilin-3 were cultured in the presence/absence of CBZ for 48 h. ER stress was measured by determining luciferase activity via the Luciferase Assay System (Promega, Madison, WI, USA) according to manufacturer’s protocol.

### 4.3. Cell Viability

Cell viability was determined using a WST-1 assay kit (Abcam, Cambridge, UK) according to manufacturer’s protocol.

### 4.4. Protein Extraction and Immunoblotting

Protein lysates were extracted, and immunoblotting was performed as outlined previously [49]. The following primary antibodies were used: FLAG M2 (Sigma Aldrich, Burlington, MA, USA), GAPDH (Sigma Aldrich, Burlington, MA, USA), PSMA1 (Abcam, Cambridge, UK). Membranes were imaged on the LI-COR^®^ Odyssey CLx Imaging System (LI-COR, Lincoln, NE, USA). Densitometric analysis of the fluorescent protein bands was presented relative to GAPDH levels. Uncropped blots can be found in Supplementary Materials Figures S1–S3.

### 4.5. RNA Extraction and Quantitative PCR (qPCR)

RNA was extracted from cells using the Reliaprep™ RNA Cell Miniprep System (Promega, Madison, WI, USA) and cDNA was synthesised from RNA using the GoScript™ Reverse Transcription System (Promega, Madison, WI, USA) according to manufacturer’s protocols. RNA was removed following the incubation with 1 U RNase H (Thermo Fisher, Waltham, MA, USA) for 20 min at 37 °C. qPCR was performed using the Power SYBR^®^ Green (Thermo Fisher, Waltham, MA, USA) method as outlined in manufacturer’s instructions on a QuantStudio 3 Real-Time PCR System (Thermo Fisher, Waltham, MA, USA). Primer sequences and cycle conditions can be found in Supplementary Materials Tables S1 and S2. Samples were analysed in triplicate and the expression was normalised to the level of *18S.* The levels of spliced *XBP1* were determined as outlined previously [9]. Briefly, the cDNA sample was amplified by 4 initial PCR cycles (95 °C for 3 min, 4 cycles of 95 °C for 40 s, 60 °C for 45 s, and 72 °C for 40 s, followed by 72 °C for 10 min) to generate double-stranded cDNA. To remove unspliced *XBP1*, the amplified cDNA was then digested using the PstI restriction enzyme (Promega, Madison, WI, USA) according to manufacturer’s instructions. This digested cDNA was then used as a template for qPCR.

### 4.6. Statistics

Results were obtained from a minimum of three individual experiments. Statistical significance was analysed by the Student’s unpaired *t*-test and statistical significance was given by *p* < 0.05.

## Data Availability

Not applicable.

## Supplementary Materials

The supporting information can be downloaded at: https://www.mdpi.com/article/10.3390/ijms24021496/s1.
