# Supplementary material for: Curcumin Reduces Pathological Endoplasmic Reticulum Stress through Increasing Proteolysis of Mutant Matrilin-3
Source: Int J Mol Sci. 2023 Jan 12;24(2):1496. doi: 10.3390/ijms24021496 (PMC9867355; doi:10.3390/ijms24021496)
Supplement: Supplementary file 1 [file ijms-24-01496-s001.zip › ijms-2134936-supplementary.pdf]

(a)

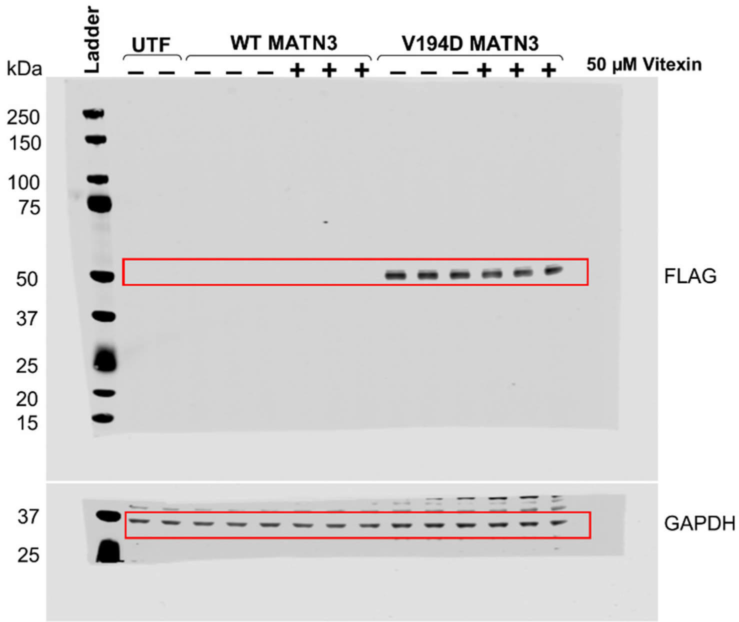

(b)

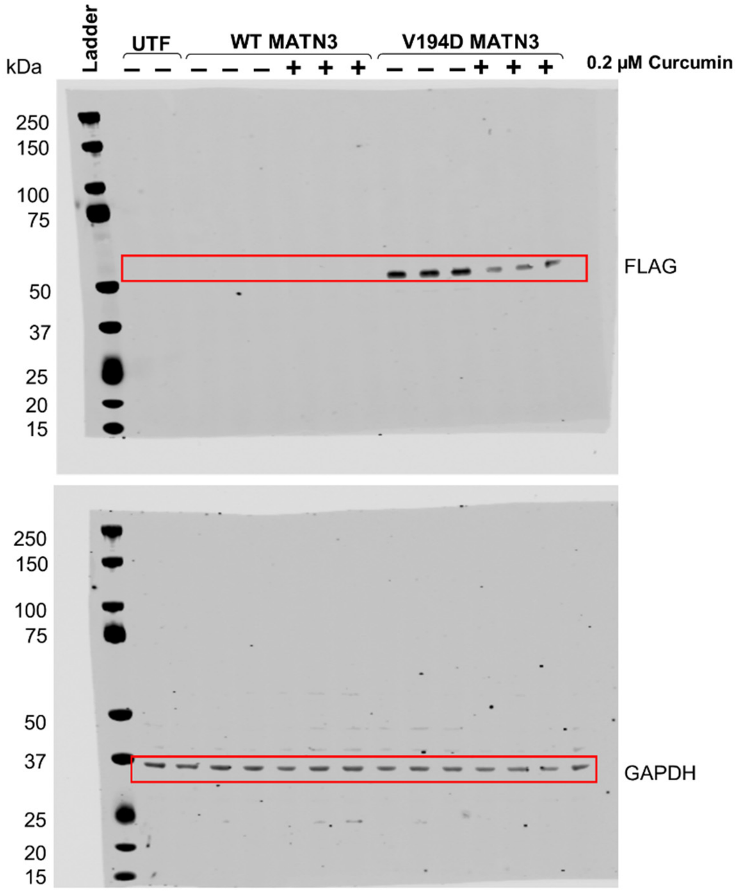

Figure S1. Curcumin treatment reduces the intracellular accumulation of mutant matrilin-3. Images of uncropped gels presented in Figure 3. Some membranes were cut prior to probing with antibodies. Cropped gels are highlighted by red box (Equal loading shown by GAPDH. FLAG: FLAG-tagged matrilin-3, GAPDH: Glyceraldehyde-3-phosphate dehydrogenase, MATN3: matrilin-3, WT: wild-type, UTF: Untransfected, n = 3).

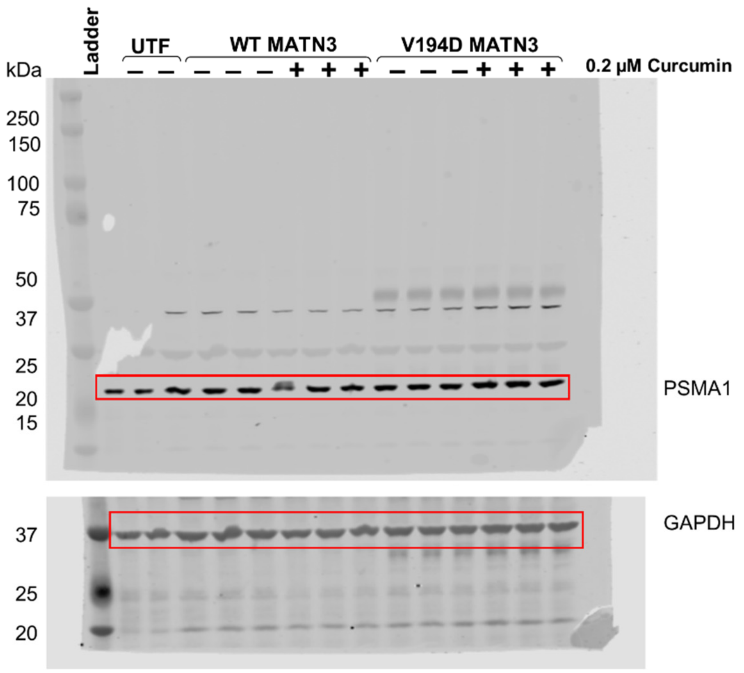

Figure S2. Curcumin treatment upregulates proteasome expression in mutant matrilin-3 expressing cells. Images of uncropped gels presented in Figure 6. Some membranes were cut prior to probing with antibodies. Cropped gels are highlighted by red box (Equal loading shown by GAPDH. PSMA1: Proteasomal subunit alpha-type 1, GAPDH: Glyceraldehyde-3-phosphate dehydrogenase, MATN3: matrilin-3, WT: wild-type, UTF: Untransfected, n = 3).

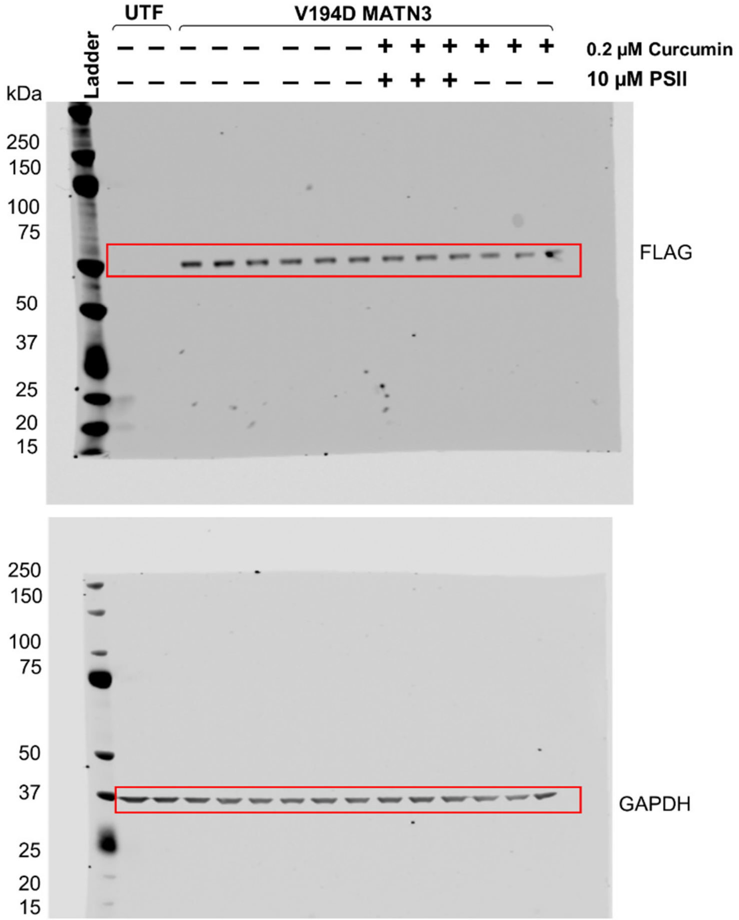

Figure S3. Curcumin promotes proteasomal degradation of V194D mutant matrilin-3 in a cell model of EDM5. Images of uncropped gels presented in Figure 7. Some membranes were cut prior to probing with antibodies. Cropped gels are highlighted by red box (Equal loading shown by GAPDH. FLAG: FLAG-tagged matrilin-3, GAPDH: Glyceraldehyde-3-phosphate dehydrogenase, MATN3: matrilin-3, PSII: Proteasome Inhibitor II, WT: wild-type, UTF: Untransfected, # significant to UTF, n = 3).

Table S1. Quantitative PCR (qPCR) primer sequences.

Gene	Primer Sequence	
	Forward	Reverse
18S	5′-GGCCCTGTAATTGGAATGAGTC-3′	5′-CCAAGATCCAACTACGAGCTT-3′
CANX	5′-GCTGGTTAGATGATGAGCCTGAG-3′	5′-ACACCACATCCAGGAGCTGAC T-3′
CRELD2	5′-AAACGGAAGATGCCTGTG-3′	5′-CTCAGGGCCACATTTTCCAC-3′
DERL1	5′-CCCAATGGACTTGGGAGGAAGA-3′	5′-GGCACACCAAATCCTGATACTCC-3′
DERL2	5′-GAAGAAGGCTCTTTCCGAGGTC-3′	5′-TCCACACATAGACGAGCATTATTG-3′
EDEM1	5′-ACGAGCAGTGAAAGCCCTTTGG-3′	5′-CCACTCTGCTTTCCAACCCAGT-3′
EDEM2	5′-GATACACAGTGGAGAAGCGAGAG-3′	5′-CAAATCCGCACTCCACCTTGCT-3′
GRP94	5′-GGAGAGTCGTGAAGCAGTTGAG-3′	5′-CCACCAAAGCACACGGAGATTC-3′
HSPA5	5′-GCTAATGCTTATGGCCTGGA-3′	5′-CGCTGGTCAAAGTCTTCTCC-3′
MANF	5′-TCACATTCTCACCAGCCACT-3′	5′-CAGGTCGATCTGCTTGTCATAC-3′
MATN3	5′-GAAGGCAAGCACCACTGTGAGT-3′	5′-GCACTCACAATGATAAGAGCCAC-3′
PDIA4	5′-CCAGCAGGTTTGATGTGAGTGG-3′	5′-GGAGACTTCTCTGACCTTGGCA-3′
PDIA6	5′-TCAGAAAGGCGAGTCTCCTGTG-3′	5′-CCTCTTGGCAATGTCCTCGTTG-3′
XBP1s	5′-GAAGCCAAGGGGAATGAAGT-3′	5′-CCAGAATGCCCAACAGGATA-3′

Table S2. Quantitative PCR (qPCR) cycling conditions.

Step	Temperature	Time
**1.**	94 °C	2 minutes
**2.**	94 °C	30 seconds
**3.**	60 °C	30 seconds
**4.**	72 °C	30 seconds
**5.**	72 °C	10 minutes
Steps 2-4 repeated x40 cycles		
**6.**	Melt curve from 55 °C to 95 °C,<br>Reading every 0.2 seconds,<br>Hold 00:00:01	
